# The Camera in Astronomy

**Published:** 1898-07

**Authors:** 


### The Camera in Astronomy.

Telescopic photography opens a wide field to the astronomer. A few women have been successful in this line, notably Mrs. Fleming, of the Harvard observatory. The minutest star-points are recorded on the sensitive plate, and hence photography is of infinite value in astronomical work.

